# Microstructure Evolution Mechanism of Ultra-Thin Dual Phase Magnesium–Lithium Alloy during Asymmetric Warm Rolling

**DOI:** 10.3390/ma15145026

**Published:** 2022-07-19

**Authors:** Shuang An, Deli Shang, Ming Chen, Cong Ma, Yanqing Lu, Xiaodong Hu

**Affiliations:** 1 School of Mechanical Engineering & Automation, University of Science and Technology Liaoning, Anshan 114000, China; 13134120916@163.com (S.A.); macong1892@163.com (C.M.); 2 State Key Laboratory of Metal Material for Marine Equipment and Application, Anshan 114000, China; 13941262991@163.com; 3 School of Materials and Metallurgy, University of Science and Technology Liaoning, Anshan 114000, China; luyanqing77@163.com (Y.L.); mgfoil@163.com (X.H.)

**Keywords:** dual phase LZ91 Mg-Li alloy, asymmetric warm rolling, FEM, VPSC, EBSD, texture evolution

## Abstract

Magnesium–lithium alloy is the lightest metal alloy material so far, and the ultra-thin plate is also one of the main trends in the future development of Mg-Li alloy. In order to explore how to prepare LZ91 ultra-thin Mg-Li alloy, this topic adopts the combination of the finite element method (FEM) and visco-plastic self-consistent (VPSC) calculation, electron back-scattered diffraction (EBSD) and tensile experiment, and uses the asymmetric warm rolling process to realize the processing of ultra-thin LZ91 Mg-Li alloy plate with a thickness of 0.25 mm. The experimental results show that the maximum basal texture strengths of 1 mm initial plate and 0.25 mm ultra-thin rolled plate are 36.02 mud and 29.19 mud, respectively. The asymmetric warm rolling process not only reduces the basal texture strength but also significantly refines the grains. The tensile strength and yield strength of 0.25 mm ultra-thin rolled plate along the rolling direction reached 206.8 MPa and 138.4 MPa, respectively. This has a positive effect on the mechanical properties of subsequent materials. VPSC results show that the base slip is the main factor in Mg-Li alloy asymmetric warm rolling, and a large number of tensile twinning are initiated due to the coordinated deformation of the body-centered cubic (BCC) phase, which is beneficial to improve the plastic deformation capacity of Mg-Li alloy.

## 1. Introduction

Advanced lightweight materials are an important core technology of “Made in China 2025”, and achieving a carbon peak is the latest goal of advanced lightweight materials technology [1]. The ultra-thin plate is one of the main trends in the development of magnesium alloys. In some highly precise electronic equipment and aerospace fields, ultra-thin magnesium alloys with a thickness of less than 0.5 mm are needed. Mg-Li alloy is the lightest metal structural material so far, which has the advantages of high specific strength, high specific stiffness, and good ductility, and has a very broad application prospect [2,3,4]. The content of Li in LZ91 Mg-Li alloy is 8.89%, which is higher than the highest solubility of Li in the α-Mg phase (Hexagonal close-packed structure, HCP) of 5.3%, forming a two-phase microstructure composed of α-Mg phase with HCP structure and β-Li phase with body-centered cubic (BCC) structure [5,6,7]. According to the Von Mises criterion, at least five independent slip systems are required to work at the same time to ensure the deformation of each grain of magnesium alloy during plastic deformation [8]. HCP is a close-packed hexagonal structure with a limited slip system, so its deformation ability is weak at room temperature. BCC is a body-centered cubic structure with 12 slip systems [9,10], which can easily meet the deformation requirements and contribute to the slip of the HCP structure. Therefore, LZ91 Mg-Li alloy has good plasticity and strength [11,12].

In order to explore how to prepare LZ91 ultra-thin Mg-Li alloy, this topic adopts the combination of simulation and experiment and uses the asymmetric warm rolling process to finally realize the processing of LZ91 Mg-Li alloy ultra-thin plate with a thickness of 0.25 mm. Generally speaking, rolling will make texture pile up and strengthen the basal texture. However, the asymmetric warm rolling experiment designed in this paper successfully processed a 0.25 mm ultra-thin plate and reduced the basal texture, which has a positive impact on the mechanical properties of subsequent materials. In order to explore how the weak basal texture of a 0.25 mm ultra-thin plate is formed, finite element method (FEM) and visco-plastic self-consistent (VPSC) were used to simulate the asymmetric warm rolling process. Combined with the opening law of slip system and twinning system and the evolution law of twinning volume fraction, describe the behavior of Mg-Li alloy in plastic deformation, reveal its plastic deformation mechanism, and provide a theoretical basis for the plastic processing of Mg-Li alloy.

## 2. Materials and Methods

### 2.1. Experiment

**Figure 1:**
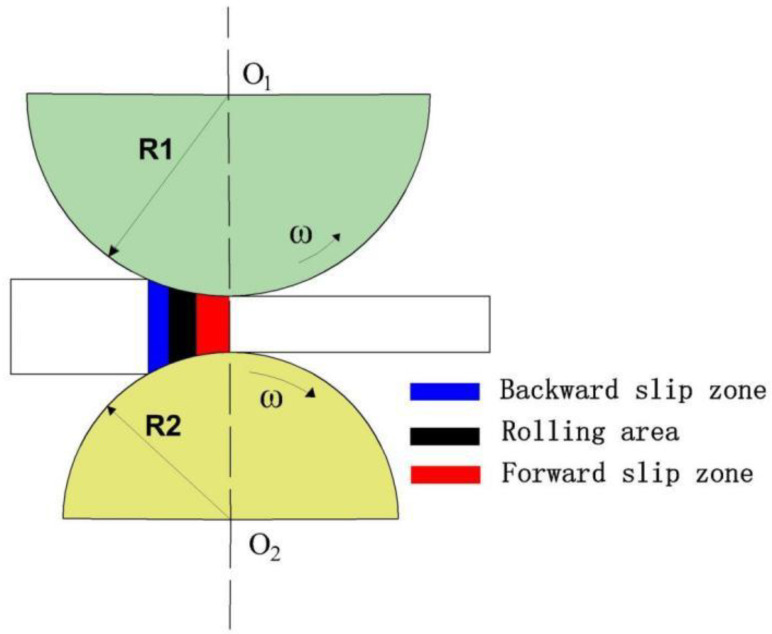
Schematic diagram of different diameter asymmetric rolling.

The material used in this paper was the LZ91 Mg-Li alloy extruded plate obtained after annealing at 200 °C for 1 h [13]. Its main chemical composition is shown in Table 1 [14]. The plate was divided by wire-cut electrical discharge machining (WEDM) for the rolling experiment. The size of the divided plate was 500 mm × 100 mm × 1 mm. The rolling equipment was a six-high warm rolling mill (University of Science and Technology Liaoning, Anshan, China), the roll temperature was 160 °C, the support roll diameter was 150 mm, the roll width was 450 mm, the rolling linear speed was 25 mm/s, the transmission ratio of the reducer was 20, the rated power of the main motor was 110 KW, and the maximum rolling force could reach 200 KN. The diameter of the upper and lower working rollers was 74 mm and 70 mm, respectively. In this experiment, multi-pass reversible rolling was used to roll the initial plate thickness from 1 mm to 0.25 mm, and the representative ultra-thin rolled plates of 0.5 mm and 0.25 mm were taken as the samples in this paper. Figure 1 is the schematic diagram of this different diameter asymmetric rolling.

The electron back-scattered diffraction (EBSD) experiment was carried out on the initial and rolled plates [15]. The steps and precautions for preparing EBSD samples are as follows [16]. Firstly, the 7 mm × 9 mm samples were prepared by WEDM. Then, rough and fine grinding the samples with the sandpaper of models 800, 1000, 1200, 1500, and 2000 successively. The polished sample surface was gently attached to the rotating velvet polishing cloth for polishing. Then electrolysis was carried out to eliminate the residual stress layer on the sample surface and improve the resolution of the EBSD experiment. The electrolyte was perchloric acid ethanol solution, and the electrolysis voltage was set to 15V. Liquid nitrogen was added to maintain the electrolyte temperature between −30 °C and −35 °C in order to obtain a better calibration effect. During the electrolytic polishing, the sample surface was connected with an anode, the stainless steel plate was connected with the cathode, and the polishing time was set at 150 s. After electrolytic polishing, the sample should be immediately put into the ultrasonic cleaning machine with acetone to remove the attachments on the sample surface. The cleaning time was 1 min, then immediately put into the sample box with acetone sealed preservation to prevent sample oxidation. Finally, the oxford instrument (equipped with an Aztec EBSD acquisition system, Oxford Instruments, Oxford, Britain) was used to analyze the EBSD characterization of the samples.

According to GB/T 228.1-2010, the room temperature tensile test was carried out, and the samples of rolled plates were prepared in the transverse direction (TD), rolling direction (RD), and 45° direction with the rolling direction, respectively, by WEDM process. The tensile test equipment was an electronic universal testing machine (Shenzhen Sansi Zongheng Technology Co., Ltd., Shenzhen, China), which was carried out at room temperature of 25 °C, and the unidirectional tensile rate is set to 1 mm/min.

### 2.2. Simulation

The asymmetric rolling of LZ91 Mg-Li alloy is simulated by FEM. According to the real size of the rolling experiment, the diameter of the upper work roll was defined as 74 mm, and the diameter of the lower work roll was defined as 70 mm. In order to reduce the simulation analysis time on the premise of ensuring the accuracy of FEM simulation results and shorten the actual plate length to stable rolling, the plate length is set to 20 mm. The roll temperature was set to 160 °C, the roll speed was set to 1rad/s, the plate rolling time was set to 1.5 s, and the mass amplification factor was 500. The FEM two-dimensional finite element model was established.

**Figure 2:**
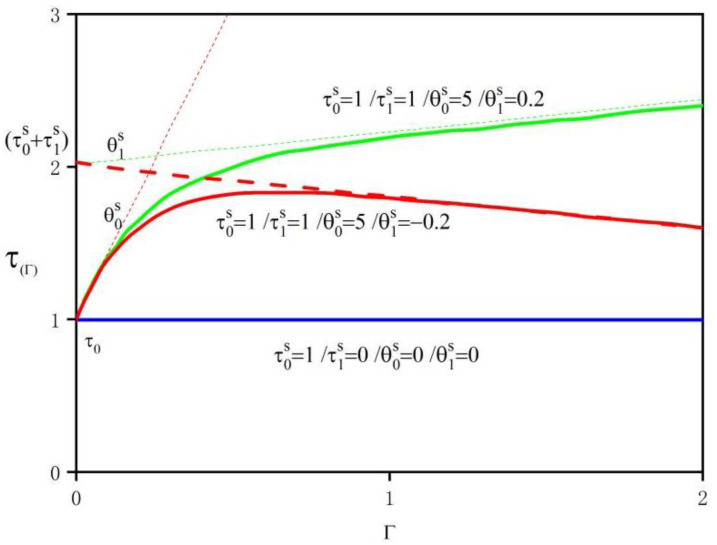
Voce hardening behavior under different parameter combinations.

Then VPSC calculation is carried out. VPSC model does not only provide material stress–strain parameters but also predicts the hardening effect, grain reorientation, and the changing trend of single grain shape during plastic deformation. The input file of the VPSC model usually consists of the following parts. In addition to the common subroutines, it also included a crystal texture file (including three Euler angles of the initial grain and the corresponding volume fraction), a single crystal property file (including the information of grain deformation mode, hardening parameters, and critical resolved shear stress (CRSS)), texture morphology file (including the shape data and shape orientation information of the initial grain) and loading process file (including information about velocity gradient, equivalent plastic strain rate, and mechanical test conditions). The critical shear stress of slip and twinning in the VPSC model was described by the hardening model, while the widely used Voce hardening model has good accuracy in texture evolution. Therefore, Voce hardening was introduced to study the plastic deformation process of materials. The critical shear stress of each slip or twin system can be expressed by cumulative shear strain [17,18], as follows:

(1) $${\hat{\tau }}^{s}=\tau_{0}^{s}+\left(\tau_{1}^{s}+\theta_{1}^{s}\Gamma \right)\left[1-exp\left(-\Gamma \left|\frac{\theta_{0}^{s}}{\tau_{1}^{s}}\right|\right)\right]$$

where $\Gamma =\sum_{s}\Delta \gamma^{s}$ is the accumulated shear strain of the slip system; $\tau_{0}^{s}$ is the initial critical shear stress CRSS; $\tau_{0}^{s}+\tau_{1}^{s}$ is the final critical shear stress CRSS; $\theta_{0}^{s}$ is the initial hardening rate; $\theta_{1}^{s}$ is the progressive hardening rate; $\theta_{0}$ and $\tau_{1}$ are generally positive. Figure 2 describes the Voce hardening behavior under several different parameter combinations and the effect of each hardening parameter on the stress–strain curve. In order to ensure the accuracy of VPSC calculation, the parameters required for modeling should be consistent with the experiment. Since the plastic deformation process was coordinated by shear, it was necessary to use the velocity gradient tensor for “polar decomposition”. Due to the linear velocity difference between upper and lower rolls in asymmetric rolling, additional shear force and shear strain are generated. The strain rate can be divided into normal strain rate and shear strain rate, and the corresponding velocity gradient tensor can be expressed as [19]:

(2) $$L={\left(\begin{array}{ccc} \dot{\varepsilon } & 0 & -\dot{\gamma }\\ 0 & 0 & 0\\ -\dot{\gamma } & 0 & -\dot{\varepsilon } \end{array}\right)}_{1-\mathrm{RD},2-\mathrm{TD},3-\mathrm{ND}}$$

The above velocity gradient expression shows that the plate is stretched in the RD direction, compressed in the ND direction, and sheared in the TD direction during the asymmetric rolling. $\dot{\varepsilon }$ is normal strain rate and $\dot{\gamma }$ is shear strain rate.

## 3. Results and Discussions

### 3.1. FEM Results Analysis

**Figure 3:**
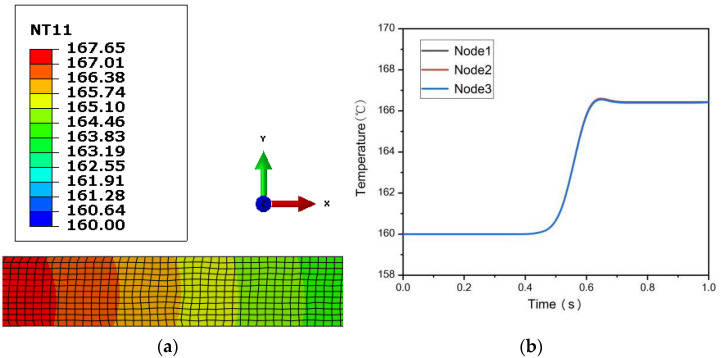
(**a**) Temperature field diagram of 0.5 mm ultra-thin rolled plate; (**b**) Temperature change curve of each monitoring point of 0.5 mm ultra-thin rolled plate.

**Figure 4:**
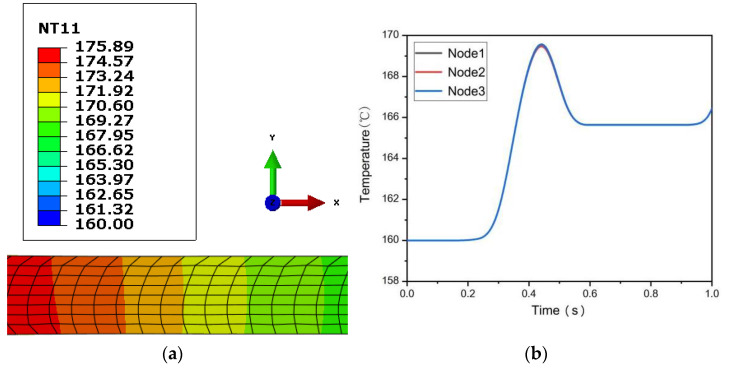
(**a**) Temperature field diagram of 0.25 mm ultra-thin rolled plate; (**b**) Temperature change curve of each monitoring point of 0.25 mm ultra-thin rolled plate.

Figure 3 and Figure 4, respectively, show the temperature field distribution of 0.5 mm and 0.25 mm ultra-thin rolled plates and the temperature change curves of three selected monitoring points when LZ91 alloy enters the steady-state stage of asymmetric warm rolling. It can be seen from the figure that the initial temperature of the two ultra-thin rolled plates before rolling deformation is 160 °C. The temperature of 0.5 mm ultra-thin rolled plate rises rapidly at a rate of 35.5 °C/s from 0.47 s until it reaches the maximum temperature of 166.6 °C at 0.65 s; the temperature rise takes 0.18 s. The temperature of the 0.25 mm rolled plate rises rapidly at a rate of 51.7 °C/s from 0.26 s until it reaches the maximum temperature of 169.6 °C at 0.44 s, and it also takes 0.18 s for the temperature rise. With the end of the rolling process and the heat exchange between the rolled plate and the surrounding medium, the temperature of the rolled plates begins to decrease. The temperature drop rate of 0.5 mm ultra-thin rolled plate is about 7.5 °C/s. When the temperature drops to 0.69 s, it gradually becomes stable and remains at about 166.3 °C. At this time, the rolled plate is separated from the roll, and the temperature inside the rolled plate is gradually transferred to the outer surface so that the temperature of each part of the rolled plate tends to be consistent. The temperature drop rate of 0.25 mm ultra-thin rolled plate is about 28.6 °C/s, and it takes only 0.14 s to drop rapidly from 169.6 °C to 165.6 °C and stabilizes for 0.36 s, and then it starts to rise up slightly. In contrast, the temperature of a 0.25 mm ultra-thin rolled plate rises and falls at a faster rate than that of a 0.5 mm rolled plate because the thinner the rolled plate is, the faster the heat energy is dissipated. Because the rolling plate is ultra-thin, the temperature field distribution in the deformation area of the asymmetric rolling is relatively uniform, and the temperature difference between the three monitoring points is also very small.

**Figure 5:**
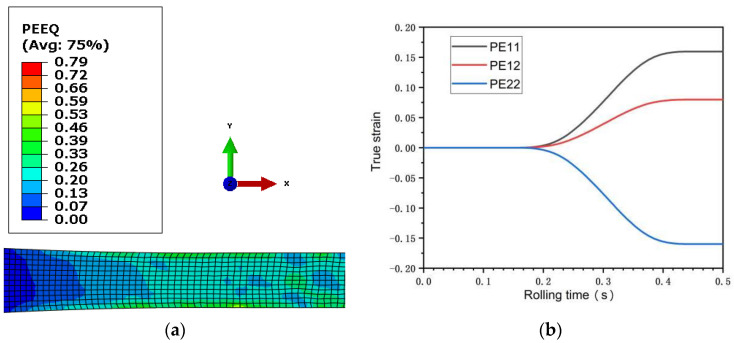
(**a**) Equivalent plastic strain field diagram of 0.5 mm ultra-thin rolled plate; (**b**) Strain curve of 0.5 mm ultra-thin rolled plate.

**Figure 6:**
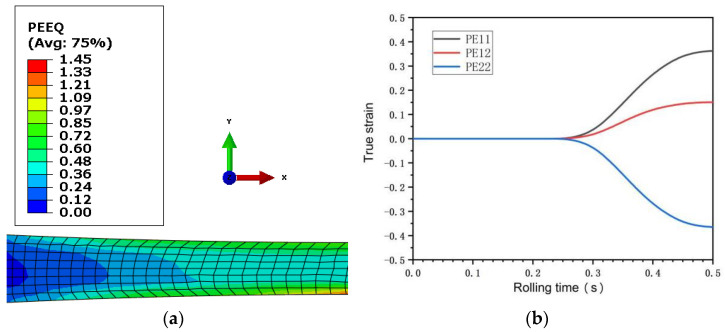
(**a**) Equivalent plastic strain field diagram of 0.25 mm ultra-thin rolled plate; (**b**) Strain curve of 0.25 mm ultra-thin rolled plate.

**Figure 7:**
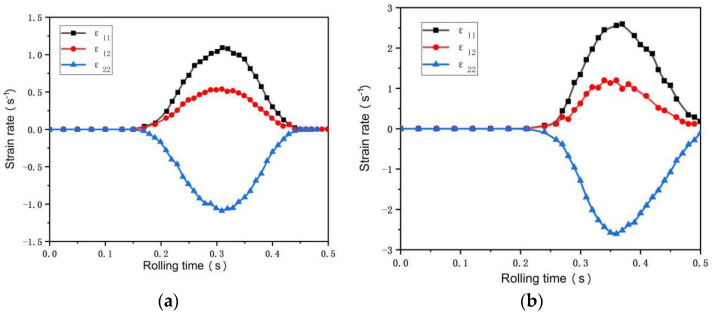
Strain rate evolution curve: (**a**) 0.5 mm ultra-thin rolled plate; (**b**) 0.25 mm ultra-thin rolled plate.

Figure 5 and Figure 6 are the equivalent plastic strain fields of asymmetric rolling of 0.5 mm and 0.25 mm ultra-thin rolled plates, respectively. PE11, PE12, and PE22 in the figure represent the normal strain, shear strain, and negative strain with the horizontal plane as the reference plane, respectively. It can be seen from the figure that the strain changes in the two sampled plates are relatively similar, and the equivalent plastic strain field presents asymmetric distribution. The interior of the 0.5 mm ultra-thin rolled plate is an uneven wave point shape, while the interior of the 0.25 mm ultra-thin rolled plate presents a stepped shape. The maximum equivalent stress values of the two rolled plates occur on the surface of the rolled plate and gradually decrease towards the inner center of the rolled plate. The maximum normal strain and maximum shear strain of 0.5 mm ultra-thin rolled plate are 0.16 and 0.08, respectively, and the maximum normal strain and maximum shear strain of 0.25 mm ultra-thin rolled plate are 0.36 and 0.15, respectively. Observed along the thickness direction of the rolled plate, the equivalent plastic strain of the lower surface is greater than that of the upper surface. Observed along the rolling direction, the equivalent plastic strain of the deformed region is larger than that of the undeformed region. The strain rate during rolling can be obtained by deriving the strain increment in Figure 5 and Figure 6 from the time increment. The strain rate changes of 0.5 mm and 0.25 mm ultra-thin rolled plates are shown in Figure 7. In the figure, $\varepsilon_{11}$, $\varepsilon_{22}$, and $\;\varepsilon_{33}$, respectively, represent the normal strain rate, shear strain rate, and negative strain rate with a horizontal plane as a reference plane. The maximum normal strain rate of 0.5 mm ultra-thin plate is 1.17 s^−1^, and the maximum shear strain rate is 0.50 s^−1^. The maximum normal strain rate of 0.25 mm plate is 2.51 s^−1^, and the maximum shear strain rate is 1.25 s^−1^. In FEM simulation, the strain rate of the sampled sheet increases first and then decreases.

### 3.2. VPSC Calculation and EBSD Experimental Results Analysis

**Figure 8:**
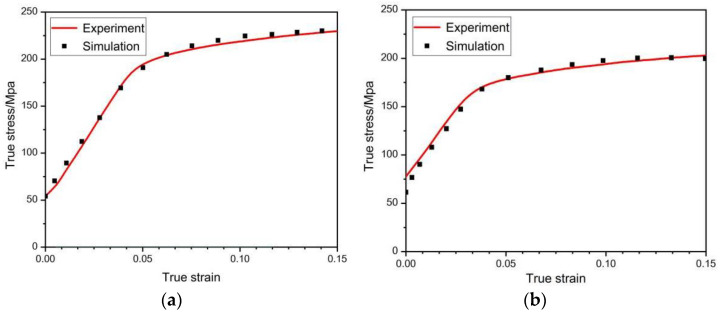
Experimental and simulation comparison of stress–strain curve: (**a**) 0.5 mm ultra-thin rolled plate; (**b**) 0.25 mm ultra-thin rolled plate.

**Figure 9:**
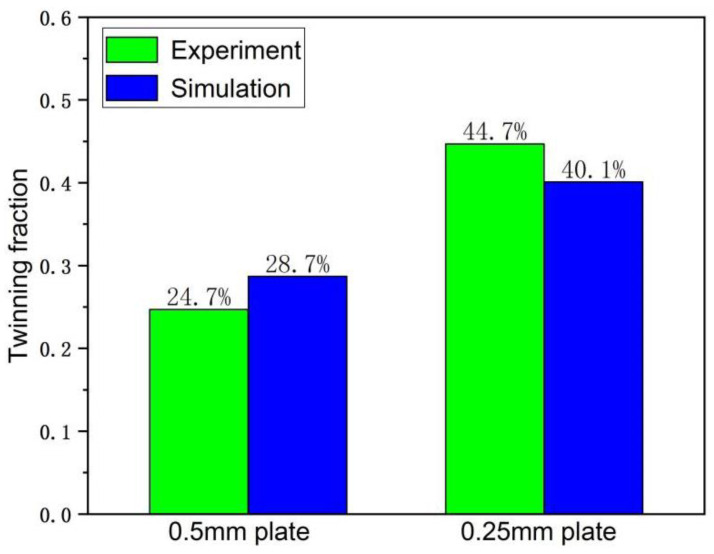
Comparison diagram of twinning area fraction of ultra-thin rolled plate obtained by VPSC calculation of asymmetric warm rolling and that obtained by EBSD experiment.

**Figure 10:**
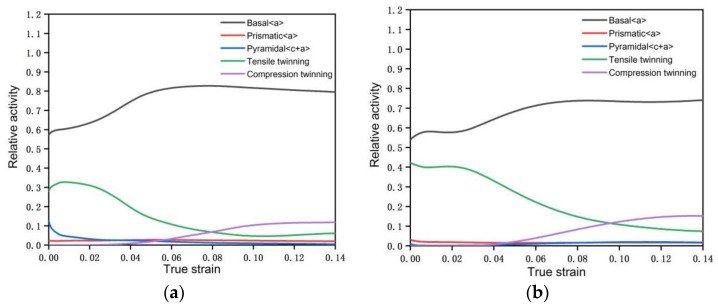
Starting amount of each slip system of LZ91 magnesium–lithium alloy sample plate: (**a**) 0.5 mm ultra-thin rolled plate; (**b**) 0.25 mm ultra-thin rolled plate.

**Figure 11:**
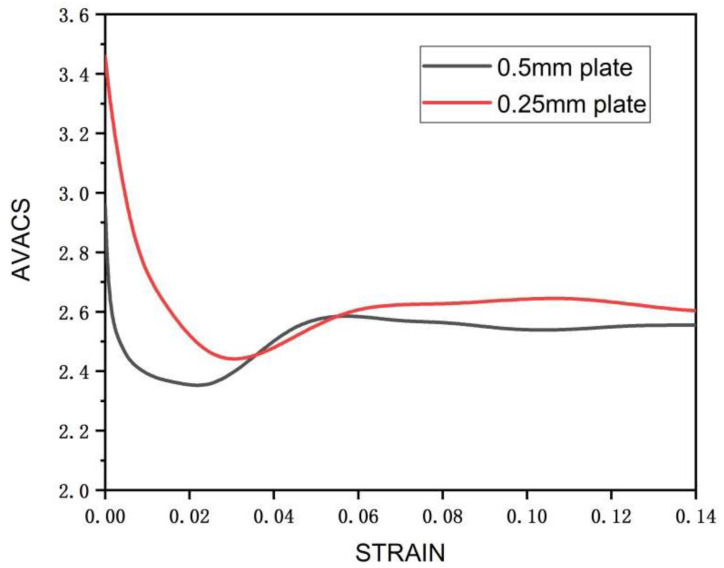
Comparison of AVACS values of 0.5 mm and 0.25 mm ultra-thin rolled plates.

Voce hardening model was used to describe the hardening of each deformation mode of the material in the process of plastic deformation. A total of 20 parameters of 5 deformation modes, including basal slip, cylindrical slip, conical slip, tensile twin, and compressive twin, were determined [20]. The final hardening parameters are shown in Table 2. Under this parameter, the stress–strain curve obtained by VPSC calculation is fitted with the real stress–strain curve of LZ91 Mg-Li alloy stretched along RD direction [21], as shown in Figure 8. The twinning area fraction of the experimental results can be obtained by counting the Euler angle data of the EBSD experiment by Image-Pro Plus 6.0 image recognition software, and the comparison with the twinning area fraction obtained by VPSC calculating of asymmetric warm rolling process is shown in Figure 9. It can be seen from Figure 9 that there is little difference between the twin area fraction of experiment and simulation, which further explains the accuracy of voce hardening parameters. Figure 10 shows the relative activation value of each slip system stretched along the RD direction. The activation of deformation mode depends on the critical shear stress value CRSS, and the total relative activation value of each slip system under each strain is 1. Studies have shown that when the deformation temperature is lower than 260 °C, the CRSS value of the non-basal slip system of magnesium alloy materials is much larger than that of the basal slip system. Therefore, at the initial stage of deformation, the plate is dominated by basal slip. Zhong et al. [22] reported that the main slip system is {110}<111> in the β phase during the rolling at room temperature. For Mg-Li alloys, the addition of Li can effectively reduce the c/a axial ratio of the α-Mg phase and activate the non-basal slip [23,24]. Due to the abundant slip system of BCC structure [25], the coordinated deformation of BCC Structure and HCP structure makes the CRSS value of tensile twins relatively low, which started in a large number. At this time, the micro deformation is supplemented by tensile twinning [26]. Rolling makes the β-Li phase and α-Mg phase gradually elongate along the RD direction [27,28], and at the interface of the two phases, the compact packing surface of the β-Li phase (011) is parallel to the compact packing surface of the α-Mg phase (0001). During the deformation process, the β-Li phase can be regarded as a sliding surface to promote the sliding of the α-Mg phase [29]. This is more conducive to the base slip but also makes the possibility of starting cylindrical slip and conical slip less. According to reference [30], the CRSS of base slip, cylindrical slip, conical slip, tensile twin, and compressive twin of LZ91 Mg-Li alloy at room temperature are 0.4 MPa, 45 MPa, 40 MPa, 3 MPa, and 28 MPa, respectively. It can be seen that twinning plays a greater role in the rolling process than cylindrical slip and conical slip. Therefore, tensile twinning should be activated to promote the plastic deformation of the LZ91 Mg-Li alloy. The average activity of the system (AVACS) represents the number of micro deformation systems per grain that participate in the calculation in the VPSC model [31]. Figure 11 shows the AVACS variation trend of 0.5 mm and 0.25 mm ultra-thin rolled plates. The AVACS value of 0.5 mm ultra-thin rolled plate is about 2.9 at the beginning of the strain and quickly decreases to the minimum of about 2.35 when the deformation reaches 0.025. With the increase in deformation, the material enters the stage of elastic–plastic deformation. The average start-up amount of the slip system increases rapidly and reaches 2.6 when the deformation is 0.05, and then slowly decreases to about 2.5 and tends to be stable. At this time, the material has completely yielded, and the number of slip systems involved in deformation gradually decreases. The AVACS of the 0.25 mm ultra-thin rolled plate has a similar variation trend to that of the 0.5 mm ultra-thin rolled plate. The AVACS reaches the maximum value of 3.45 at the beginning of deformation and decreases to the minimum value of 2.45 when the deformation reaches 0.003 and then enters the elastic–plastic deformation stage. The AVACS increases slowly and finally stabilizes at about 2.6 until the material is completely yielded.

**Figure 12:**
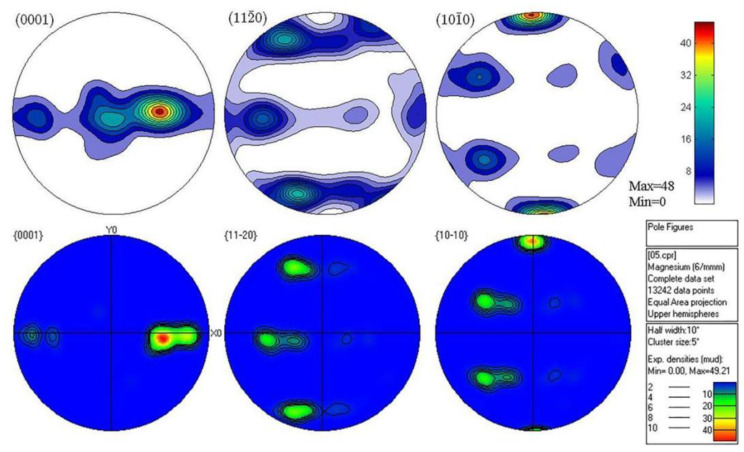
Comparison of pole diagram between VPSC calculation and EBSD experiment of 0.5 mm ultra-thin rolled plate.

**Figure 13:**
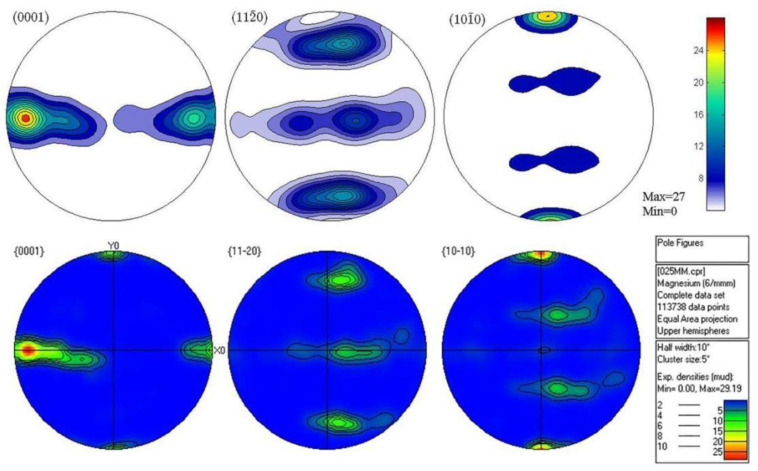
Comparison of pole diagram between VPSC calculation and EBSD experiment of 0.25 mm ultra-thin rolled plate.

**Figure 14:**
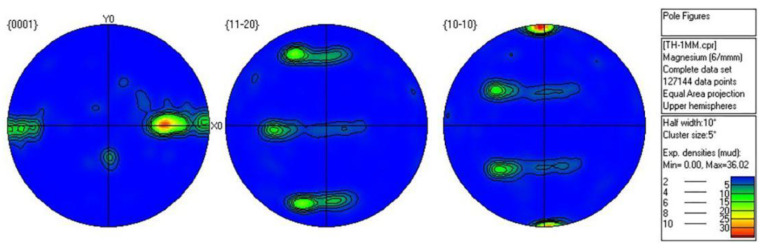
EBSD experimental polar diagram of 1 mm initial plate.

The texture output of pole diagram was obtained by processing the texture data coupled with EBSD channel, and EBSD measurements clearly brought out the HCP α-phase, whereas the BCC β-phase was not amenable for detection in EBSD. Therefore, the analysis performed here was purely based on the characterization of α-phase only [32]. Figure 12 and Figure 13 are the comparison diagrams of the VPSC calculated pole diagram and EBSD experimental pole diagram of 0.5 mm and 0.25 mm ultra-thin rolled plate simulated according to the hardening parameters in Table 2. It is obvious from the figure that the simulation results are consistent with the experimental results, which further proves the accuracy of the simulation parameters. Figure 14 shows the EBSD experimental polar diagram results of 1 mm initial plate. The maximum value of basal texture is 36.02 mud. When rolled to 0.5 mm, the maximum basal texture is significantly increased compared with that of the initial plate, reaching 49.21 mud. This is because the CRSS value of base slip is obviously lower than that of twin, cylindrical, and conical slip systems at the initial stage of asymmetric rolling deformation. Therefore, the formation of the texture of LZ91 Mg-Li alloy is mainly affected by base slip, and a large number of activation of base slip leads to strong base texture during rolling. When rolling to 0.25 mm, the maximum value of basal texture decreased to 29.19 mud and deflected 30° towards the rolling direction. This may be due to the reverse friction caused by the linear velocity difference between the upper and lower rolls, forming a “rubbing zone”. Due to the influence of shear force, the grain orientation in the rubbing zone becomes more random, so the anisotropy of the base surface is weakened, and the texture strength of the base surface is reduced. The reduction in basal texture has a positive effect on the mechanical properties of subsequent materials. From the starting amount of each slip system in VPSC results, it can be seen that the contribution value of tensile twinning of 0.25 mm ultra-thin rolled plate is larger, and a large amount of tensile twinning and the weakened basal texture promotes the plastic deformation ability of the material. Due to the asymmetric warm rolling process, the tensile stress along the rolling direction and the compressive stress along the normal direction of the rolling surface is introduced into the ultra-thin rolling plate, which not only leads to the transformation of the texture of the ultra-thin Mg-Li alloy rolling plate but also leads to the more uniform distribution of the second phase grains in LZ91 Mg-Li alloy, which can effectively enhance the comprehensive mechanical properties of the material.

**Figure 15:**
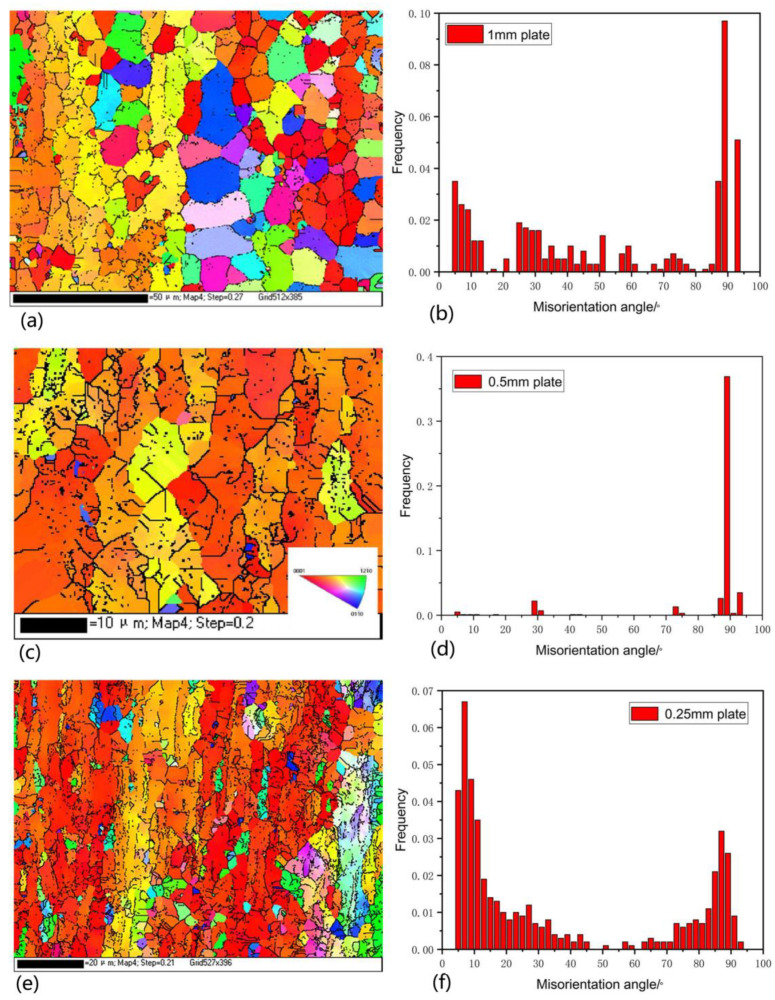
(**a**) IPF diagram of 1 mm initial rolled plate; (**b**) Orientation difference angle distribution diagram of 1 mm initial plate; (**c**) IPF diagram of 0.5 mm ultra-thin rolled plate; (**d**) Orientation difference angle distribution diagram of 0.5 mm ultra-thin rolled plate; (**e**) IPF diagram of 0.25 mm ultra-thin rolled plate; (**f**) Orientation difference angle distribution diagram of 0.25 mm ultra-thin rolled plate.

Figure 15a–f are the IPF diagrams and orientation difference angle distribution diagrams of 1 mm, 0.5 mm, and 0.25 mm samples, respectively. It can be seen from Figure 15a that the microstructure of the initial plate is composed of grains with different crystal orientations [33]. Consistently, Kumar et al. [34] reported that EBSD measurements were only possible for the α phase, where the β phase of all the samples was not detected due to the oxide formation on its surface. It can be seen from the orientation difference angle distribution diagram that the tensile twin {10–12} of 1 mm initial plate reaches the peak at the orientation difference angle of 89°, and the statistical volume fraction of tensile twin initiation is 0.097. At the same time, the volume fraction at 93° also reaches 0.051, while the volume fractions of compression twin {10–11} and {10–13} are only 0.02 and 0.017, and the volume fractions of twin {10–11}-{10–12} and {10–13}-{10–12} are 0.035 and 0.042, respectively. When rolling to 0.5 mm, the tensile twin {10–12} has a strong peak value of 0.369 volume fraction at the orientation difference angle of 89°. Combined with the IPF diagram, it can be seen that there are not only red tensile twins parallel to the normal direction of the rolled plate but also many red grains, which should be twin products. This further confirms the simulation results of VPSC that stretch twinning is significantly activated to coordinate deformation. The starting values of compression twins {10–11} and {10–13} of 0.5 mm ultra-thin rolled plate are zero, and the double twins {10–11}-{10–12} and {10–13}-{10–12} are only 0.001. When the rolling is continued to 0.25 mm, it can be seen from the IPF diagram that the plate structure is obviously refined. At this time, the volume fraction difference between the peak value of the stretching twin {10–12} starting amount and the nearby orientation difference angle is not as obvious as that of a 0.5 mm ultra-thin rolling plate. When the orientation difference angle is 87°, it reaches the peak value of 0.032, and the total volume fraction of statistical tensile twins is 0.099. Compared with a 0.5 mm ultra-thin rolled plate, the volume fraction of tensile twin initiation decreases by 73%, which may be that the twinned matrix presents an orientation parallel to the normal direction of the rolled plate. This orientation can neither slip the base plane nor tensile twins in the subsequent rolling strain, but compression twins may occur [24]. At this time, the volume fractions of compression twins {10–11} and {10–13} are 0.003 and 0.01. The volume fraction of double twins {10–11}-{10–12} and {10–13}-{10–12} are 0.021 and 0.062, respectively. Although the twin volume fraction calculated by EBSD is only the volume fraction of the twin boundary left under the corresponding rolling state, rather than the volume fraction of tensile twin products in the whole rolling process, these statistics are still very valuable [35].

### 3.3. Tensile Results Analysis

Table 3 shows the mechanical properties of the sampled plates. The maximum yield strength of 0.5 mm ultra-thin rolled plate reaches 165.5 MPa when it is stretched along the RD direction. According to the polar graph analysis, the strong basal texture is not conducive to basal slip, which leads to high yield strength when stretched along the RD direction. The tensile strength and yield ratio in the RD direction are also the largest, 237.4 MPa and 0.70, respectively. The yield strength of 0.25 mm ultra-thin rolled plate stretched along RD direction is 138.4 MPa, which is lower than that of 0.5 mm ultra-thin rolled plate, and the anisotropy of yield strength is also significantly improved. This is mainly due to the weakening and bias of the base texture, which increases the grain composition with a large deviation from the normal angle of the plate, effectively improves the base slip of the plate at room temperature, and reduces the yield strength of the plate. For dual-phase Mg-Li alloy, the presence of the BCC structure would significantly reduce the mechanical anisotropy [36] because there are 12 slip systems for {110}<111> slip of β phase, which could satisfy the Von Mises criterion for strain compatibility well. The maximum tensile strength of a 0.25 mm ultra-thin rolled plate in the 45° direction is 208.7 MPa, which is also lower than that of a 0.25 mm ultra-thin rolled plate. The elongation of 0.5 mm and 0.25 mm ultra-thin rolled plates is the best in the 45° direction, which is 51.51% and 34.36%, respectively. The reason for the high elongation of LZ91 alloy is the coordinated deformation of the HCP phase and BCC phase. According to reference [37], the hardness of the β-Li phase is higher than that of α-Mg. During the tensile process, the β-Li phase and α-Mg phase are not deformed at the same time. The soft β-Li phase deforms preferentially, and to a certain extent, then the hard α-Mg phase begins to deform. In other words, the softer β-Li phase deforms first, while the harder α-Mg phase still maintains elastic deformation. With the continuous deformation of the β-Li phase, the stress is transferred to the α-Mg phase. When the stress is large enough to reach the elastic limit of the α-Mg phase, the α-Mg phase begins to plastic deformation, and then the coordinated deformation of the two phases occurs. At the same time, the interface between the two phases has a great hindrance to dislocation and crack propagation. The addition of Li improves the ductility of magnesium alloy [38,39], so the dual-phase LZ91 Mg-Li alloy has excellent plasticity and tensile forming properties at room temperature.

## 4. Conclusions

In order to explore how the weak basal texture of a 0.25 mm ultra-thin plate is formed, this paper uses FEM and VPSC calculation, combined with EBSD and room temperature tensile experiment, to study the effect of microstructure evolution on texture and mechanical properties of LZ91 dual-phase Mg-Li alloy plates during the asymmetric warm rolling. The main conclusions are as follows:

FEM simulation results show that the equivalent plastic strain of the lower surface is larger than that of the upper surface because the rolling force is unevenly distributed along the thickness direction of the plate, but the plastic strain difference between the upper and lower surfaces is very small because the plate is too thin. The strain rate variation of 0.5 mm and 0.25 mm ultra-thin plates is the same, which increases first and then decreases.

The highest tensile strength, yield strength, yield ratio, and elongation of 0.5 mm rolled plate are 165.5 MPa, 237.4 MPa, 0.70, and 51.51%, respectively. The highest tensile strength, yield strength, yield ratio, and elongation of 0.25 mm ultra-thin rolled plate are 138.4 MPa, 208.7 MPa, 0.76, and 34.36%, respectively. Compared with the 0.5 mm rolled plate, the tensile strength, yield strength, and elongation of the 0.25 mm ultra-thin rolled plate all decreased, but the performance is still good, which provides the possibility for subsequent processing.

VPSC calculation results show that the base slip is dominant in the LZ91 dual-phase Mg-Li alloy ultra-thin plate, but the coordinated deformation of the β-Li phase and α-Mg phase initiates a large number of tensile twinning. At the same time, it can also be seen from the IPF diagram of the EBSD experiment that tensile twinning and many red grains appear in the rolled plates, indicating that tensile twinning is significantly activated to coordinate deformation, which further proves that twinning can effectively promote the plastic deformation of LZ91 Mg-Li alloy during asymmetric warm rolling.

According to the EBSD experimental results, the maximum basal texture strength of 1 mm initial plate is 36.02 mud, and that of a 0.25 mm ultra-thin rolled plate is 29.19 mud. The results indicate that the asymmetric warm rolling process can be considered when preparing ultra-thin Mg-Li alloy plates because this processing method can effectively weaken the basal texture and improve its plastic deformation ability. Therefore, this project provides more possibilities for the preparation and processing of ultra-thin LZ91 Mg-Li alloy.

## Figures and Tables

**Table 1:** Chemical composition of LZ91 magnesium–lithium alloy.

Alloying Element	Li	Zn	Mn	Si	Fe	Cu	Ni	Mg
Content (wt.%)	9.0200	0.9630	0.0160	0.0051	0.0035	0.0018	0.0002	balance

**Table 2:** The best fitting hardening parameters of VPSC calculation asymmetric warm rolling process.

Rolling	Deformation Mode	$\tau_{0}(\mathrm{MPa})$	$\tau_{1}(\mathrm{MPa})$	$\theta_{0}(\mathrm{MPa})$	$\theta_{1}(\mathrm{MPa})$
0.5 mm	Basal<a>	12	40	600	1
	Prismal<a>	14	80	1000	2
	Pyramidal<c+a>	18	220	2500	1
	Tensile twinning	13	50	600	6
	Compression twinning	30	80	2900	10
0.25 mm	Basal<a>	18	21	450	8
	Prismal<a>	35	33	680	8
	Pyramidal<c+a>	46	55	1500	9
	Tensile twinning	30	20	480	12
	Compression twinning	70	30	2250	20

**Table 3:** Mechanical properties of LZ91 Mg-Li alloy plate.

Tensile Samples		Yield Strength $\sigma_{s}/\mathrm{MPa}$	Tensile Strength $\sigma_{b}/\mathrm{MPa}$	Elongation δ/%	Yield Ratio $\sigma_{s}$$/\sigma_{b}$
0.5 mm	RD	165.5	237.4	28.00	0.70
	45°	128.2	214.4	51.51	0.60
	TD	138.4	200.5	18.13	0.69
0.25 mm	RD	138.4	206.8	24.68	0.67
	45°	106.9	208.7	34.36	0.51
	TD	137.1	179.8	13.97	0.76

## Data Availability

Not applicable.
